# Expression, Purification and Characterization of the Hepatitis E Virus Like-Particles in the *Pichia pastoris*

**DOI:** 10.3389/fmicb.2020.00141

**Published:** 2020-02-06

**Authors:** Jyoti Gupta, Sheetal Kaul, Akriti Srivastava, Neha Kaushik, Sukanya Ghosh, Chandresh Sharma, Gaurav Batra, Manidipa Banerjee, Shalimar, Baibaswata Nayak, C. T. Ranjith-Kumar, Milan Surjit

**Affiliations:** ^1^Virology Laboratory, Vaccine and Infectious Disease Research Centre, Translational Health Science and Technology Institute, NCR Biotech Science Cluster, Faridabad, India; ^2^International Centre for Genetic Engineering and Biotechnology, New Delhi, India; ^3^Centre for Bio-Design and Diagnostics, Translational Health Science and Technology Institute, NCR Biotech Science Cluster, Faridabad, India; ^4^School of Life Sciences, Manipal University, Manipal, India; ^5^Kusuma School of Biological Sciences, Indian Institute of Technology Delhi, New Delhi, India; ^6^Department of Gastroenterology, All India Institute of Medical Sciences, New Delhi, India; ^7^University School of Biotechnology, Guru Gobind Singh Indraprastha University, New Delhi, India

**Keywords:** viral hepatitis, hepatitis E virus, open reading frame 2, virus-like particle, *Pichia pastoris*

## Abstract

Hepatitis E virus (HEV) is associated with acute hepatitis disease, which may lead to chronic disease in immunocompromised individuals. The disease is particularly severe among pregnant women (20–30% mortality). The only licensed vaccine against HEV, which is available in China, is the *Escherichia coli* purified recombinant virus-like particles (VLPs) encompassing the 368–660 amino acids (aa) of the viral ORF2 protein. The viral capsid is formed by the ORF2 protein, which harbors three glycosylation sites. Baculo virus expression system has been employed to generate a glycosylated VLP, which encompasses 112–608aa of the ORF2 protein. Here, we sought to produce a recombinant VLP containing 112–608aa of the ORF2 protein in *Pichia pastoris (P. pastoris)* expression system. The cDNA sequence encoding 112–608aa of the ORF2 protein was fused with the α-mating factor secretion signal coding sequence (for release of the fusion protein to the culture medium) and cloned into the yeast vector pPICZα. Optimum expression of recombinant protein was obtained at 72 h induction in 1.5% methanol using inoculum density (A_600_) of 80 and at pH-3.0 of the culture medium. Identity of the purified protein was confirmed by mass spectrometry analysis. Further studies revealed the glycosylation pattern and VLP nature of the purified protein. Immunization of BALB/c mice with these VLPs induced potent immune response as evidenced by the high ORF2 specific IgG titer and augmented splenocyte proliferation in a dose dependent manner. 112–608aa ORF2 VLPs produced in *P. pastoris* appears to be a suitable candidate for development of diagnostic and prophylactic reagents against the hepatitis E.

## Introduction

Hepatitis E virus (HEV) is a single-stranded, positive-sense RNA virus with a size of 27–34 nm, belonging to the family *Hepeviridae*. It is a major cause of acute viral hepatitis (Smith et al., 2014; Nan and Zhang, 2016). HEV is responsible for outbreaks and sporadic cases in both developing and developed countries. The disease is self-limiting and mostly resolve after the acute phase but can progresses to chronic hepatitis in some cases (Kamar et al., 2008; Purcell and Emerson, 2008). The mortality rate ranges from 0.5 to 3% in young adults and increases up to 30% in pregnant women (Chaudhry et al., 2015). The HEVs are classified into seven genotypes. Genotype 1and 2 viruses exclusively infect humans and no animal reservoir is yet known. Genotype 3 and genotype 4 are highly diverse and zoonotic with an expanded host range. Genotype 5 and genotype 6 viruses predominately infect wild boar whereas, genotype 7 viruses infect camel. All the genotypes are antigenically conserved and there is only one serotype, making the development of a univalent hepatitis E vaccine reasonable (Schlauder and Mushahwar, 2001; Emerson et al., 2006; Lu et al., 2006).

The HEV genome is approximately 7.2 kb and has three open reading frames (ORFs). ORF1 encodes a non-structural polyprotein with seven distinct domains: methyltransferase, Y-domain, papain-like cysteine protease, V domain, macrodomain, helicase, and RNA dependent RNA polymerase. ORF1 is followed by ORF2, which encodes the capsid protein, and ORF3, which overlaps with ORF2 and encodes a phosphoprotein that modulates host cellular activities and plays a role in release of the progeny virions (Mori and Matsuura, 2011; Nan and Zhang, 2016). HEV genotype 1 (G1-HEV) has a fourth ORF, which encodes the ORF4 protein that plays an essential role in viral replication (Nair et al., 2016). The ORF2 is a 660aa protein, which has three domains [shell (S), middle (M), and protruding (P)] (Cao and Meng, 2012) and three N-linked glycosylation sites (Zafrullah et al., 1999). Immune dominant epitopes of ORF2 protein are conserved among all HEV genotypes against which all neutralizing antibodies are targeted (Lu et al., 2006). Therefore, efforts to develop a safe and effective vaccine against HEV have focused on ORF2 protein. Homo-oligomerization ability of the ORF2 protein has been utilized to generate virus-like particles (VLPs), *in vitro* (Li et al., 2005a; Roldao et al., 2010).

Virus-like particles express viral antigen and epitopes on their surface, which may provide strong and long-lasting humoral and cellular immune responses. However, they lack viral genetic material. Therefore, VLPs may be a safe and effective strategy for vaccine development against viral diseases (Murata et al., 2003; Crisci et al., 2012; Syomin and Ilyin, 2019). Cervarix (Glaxosmithkline, United Kingdom), Gardasil and Gardasil9 (Merck, United States) are commercially available VLP-based vaccines against the HPV. Similarly, Engerix (Glaxosmithkline, United Kingdom), Recombivax HB (Merck, United States) and Sci-B-Vac (VBI Vaccines, United States) are commercially available VLP-based vaccines against the HBV. Further, VLP-based vaccines against the hepatitis C virus (HCV) and the human immunodeficiency virus (HIV) have generated promising results in preclinical studies (Murata et al., 2003; Olsson et al., 2007; Zhao et al., 2016).

In the case of HEV, different regions of the viral capsid protein have been expressed in bacteria, yeast and insect cell culture system (baculovirus/insect cells) to generate VLPs (Robinson et al., 1998; Li et al., 2005b, c; Simanavicius et al., 2018). The 368–606aa region of the ORF2 protein has been purified from the insoluble fraction of *Escherichia coli (E. coli)*, which assembles into VLPs, *in vitro* (Zhao et al., 2013; Wei et al., 2014). This VLP offers 100% efficacy in clinical trial against symptomatic hepatitis E and it is licensed for commercial use as a vaccine in China (Zhu et al., 2010; Li et al., 2015). Other smaller peptides such as E2 (394–606), E2s (459–606), which carry neutralizing epitopes, have been expressed in *E. coli.* These peptides also form VLPs, which show immunogenicity in primates (Li et al., 2005b, 2009; Zhang et al., 2005). By using baculovirus vectors, two variants of the ORF2 protein (56 kDa and 53 kDa) were purified from the insect cell line, of which the 53 kDa protein could self-assemble into VLPs that were slightly smaller than the native HEV particles and these proteins exhibited immunogenicity and protective efficacy in HEV challenged Rhesus monkeys (Tsarev et al., 1997; Guu et al., 2009; Xing et al., 2010). Further analysis of the ORF2 truncations revealed that removal of 111aa from the N-terminus and 52aa from the c-terminus (112–608) of G1-HEV ORF2 protein substantially enhanced VLP formation in insect cells and produced *T* = 1 VLP similar to the native virion (Li et al., 1997, 2004; Xing et al., 2010). The 112–608aa VLP exhibits all immunodominant neutralization epitopes and generates efficient humoral response in primate models (Khudyakov et al., 1999; Zhang M. et al., 2001; Li et al., 2004, 2011; Xing et al., 2010). The baculovirus-expressed N-terminally truncated rat HEV-3 capsid protein formed VLP of 35 nm in diameter, similar to native HEV particles having no RNA packaging inside and formed *T* = 1 virion (Yamashita et al., 2009). Compared to the baculovirus expression system, the yeast (*Pichia pastoris*) expression system has the advantage of ease of manipulation, high yield, and low production cost. *P. pastoris* has been successfully used for vaccine production against viruses such as hepatitis B virus (HBV), Coxsackie virus and human enterovirus 71 (Cregg et al., 1987; Wang et al., 2013; Zhang et al., 2016). In an earlier study, 382–674aa region of the capsid protein of HEV (named as p293 ORF2) was expressed in the *P. pastoris* as a His-tagged fusion protein. The secreted p293 ORF2 was purified from the culture supernatant and analyzed by electron microscopy, which revealed it to be assembled into VLPs of 30nm size (Yang et al., 2010).

In the present study, we expressed 112–608aa region of the ORF2 protein of g1-HEV in *P. pastoris as an* N-terminal His-tag fusion protein. ORF2 was secreted to the culture medium as an N-linked glycoprotein, which was purified by Ni-NTA affinity chromatography, followed by density gradient centrifugation. The purified protein was characterized and its immunogenicity was evaluated in mice. *P. pastoris* expression system appears to be a better alternative to the baculovirus expression system for production of 112–608aa VLP.

## Materials and Methods

### Cloning and Generation of *Pichia* Transformant Containing pPICZαA-ORF2

The G1-HEV ORF2 region (112–608aa) was amplified from pSKHEV2 by PCR using the following forward and reverse primers: 5′AGCCGCGGCGGCCGCGCGGTCGCTCCGGC-3′ and 5′CATTGTTCTAGAAATGCTAGCACAGAGTGG3′. The PCR product was digested with *Not*I and *Xba*I restriction enzymes and ligated into the pPICZα vector predigested with the same enzymes. The resulting construct was named as pPICZα 112–608aa ORF2. The clone was confirmed by sequencing of the insert. pPICZα and pPICZα 112–608aa ORF2 vectors were linearized with *Bst*XI enzyme and electroporated into competent *P. pastoris* strain KM71H (Thermo Fisher Scientific, Massachusetts, United States). Transformants were grown on YPDS (1% yeast extract, 2% peptone, 2% dextrose, and 1M sorbitol) plates containing 100 μg/ml zeocin and incubated at 30°C in a humidified incubator. Single colonies from pPICZα and pPICzα 112–608aa ORF2 transformants, were inoculated in YPDS medium and incubated in a rotatory shaker (270 rpm) for a period of 16–18 h at 28.5°C till the absorbance (A_600_) reached ∼ 2.0, followed by inoculation in BMGY (1% yeast extract, 2% peptone, 100 mM phosphate buffer, 1.34% Yeast nitrogen base, 0.02% Biotin, 1% glycerol) media. The culture was grown for a period of 16–18 h under similar conditions till the A_600_ reached ∼ 16.0. The culture was centrifuged and the pellet was re-suspended and grown in BMMY (1% yeast extract, 2% peptone, 100 mM phosphate buffer, 1.34% Yeast nitrogen base, 0.4 μg/mL Biotin) till A_600_ was ∼ 60–70. 1.5% methanol was added at 24 h interval till 72 h and culture was grown at 28.5°C, 270 rpm. The culture was centrifuged in a SW28 rotor in an ultracentrifuge (Beckman Coulter, Indianapolis, IN, United States) at 125,000 × *g* and medium was collected. Presence of ORF2 protein in the culture supernatant was detected by enzyme linked immunosorbent assay (ELISA), SDS-PAGE Coomassie blue staining and Western blot using anti-ORF2 antibody.

### Enzyme Linked Immunosorbent Assay (ELISA)

Ninety-six well microtiter plates (Nunc, Thermo Fisher Scientific, Massachusetts, United States) were coated with 5 μl of culture supernatant mixed with 95 μl of 100 mM sodium bicarbonate buffer (pH 9.6) and kept at 4°C overnight. The plates were washed thrice in 200 μl/well of wash buffer (PBS + 0.1% Tween20, pH 7.4) and blocked with 200 μl/well of blocking buffer (PBS + 1% BSA) at 37°C for 2 h. Subsequently, plates were washed with wash buffer and incubated with anti-ORF2 rabbit polyclonal antibody (Nair et al., 2016) at a dilution of 1:1000 in assay buffer (PBS + 0.1% Tween20, 0.2% BSA) at 37°C for 2 h. Next, plates were incubated with HRP conjugated anti-rabbit IgG in assay buffer for 1 h at 37°C and washed three times in wash buffer. HRP activity was measured by colorimetry using TMB 3,3′,5,5′-tetramethylbenzidine, (Sigma, St. Louis, MO, United States) as the substrate. Values were measured at A_450_ using a multimode microplate reader (Synergy HT, BioTek, Vermont, United States).

### SDS-PAGE and Western Blot Analysis

The protein samples were mixed with 2X Laemmeli buffer (50 mM Tris–HCl,100 mM dithiothreitol, 4% SDS, 0.2% bromophenol blue, and 20% glycerol), incubated for 5 min at 95°C and resolved on 10% SDS-PAGE gels. For the western blot analysis, the proteins were transferred to 0.2 μm polyvinylidene fluoride (PVDF) membrane (Pall Corporation, New York, NY, United States). The membrane was incubated in blocking buffer (5% BSA in PBS) for 1 h at room temperature and then incubated in anti-ORF2 polyclonal rabbit antibody diluted 1:1000 in buffer I [Phosphate buffer saline (PBS) + 0.1% Tween20 + 5% BSA] at 4°C overnight. The membrane was washed thrice with wash buffer (PBS + 0.1% Tween 20) and incubated with HRP conjugated goat anti rabbit antibody diluted 1:5000 in buffer I for 1 h at 25°C. After 3 times washing with wash buffer, blot was developed using a chemiluminiscence Substrate (Bio-Rad, California, United States).

### Protein Purification by Immobilized Metal Affinity Chromatography

The culture medium was harvested by centrifugation at 7800 × *g* for 1 h, supernatant was mixed with equilibration buffer [5 mM Imidazole, 50 mM Tris–Cl (pH 7.5), 500 mM NaCl] containing 1 mM phenyl methyl sulfonyl fluoride (PMSF) and incubated with Ni-Agarose beads for 2 h. Washing was done with wash buffer (50 mM Imidazole in equilibration buffer) followed by elution of the bound proteins in 250 mM and 500 mM imidazole. For large scale purification, the culture supernatant was equilibrated with equilibration buffer (as mentioned above), loaded on to HisTrap FF Ni-Sepharose column (GE Healthcare, Illinois, United States) fitted to a FPLC (Fast protein liquid chromatography) system (AKTA purifier, GE healthcare, Illinois, United States). Washing was done in 4–50 mM imidazole gradient, followed by elution of the bound protein in a gradient of 50–500 mM imidazole. The eluted fractions showing protein peaks were analyzed by gel electrophoresis. The ORF2 containing protein fractions were pooled, concentrated and buffer exchanged to PBS using a 10 kDa centrifugal filter device (Pall Corporation, New York, NY, United States).

### Mass Spectrometry

The ∼56 kDa band of protein was gel excised, placed in 1.5 ml microtube and centrifuged at 10,000 rpm for 5 min. 100 μl of destaining solution (1:1 ratio if 100 mM ammonium bicarbonate and 100% acetonitrile) was added and incubated for 30 min, centrifuged at 5000 rpm for 1 min at room temperature. The shrinked gel pieces were dried in speed-vac for 15 min at 30°C, mixed with trypsin buffer (13 ng/μl Trypsin, 10 mM ammonium bicarbonate + 10% acteonitrile) and kept on ice. After addition of trypsin buffer, pH was checked and 100 mM ammonium bicarbonate was added to obtain pH 7.0, followed by 90 min incubation on ice for 1 h, followed by incubation at 37°C overnight. Next day, the sample was centrifuged at 10,000 rpm for 1 min. The supernatant was collected and mass spectrometry was performed by MALDI MS-MS at the “Advanced instrumentation research facility” (Special Centre for Molecular Medicine, Jawaharlal Nehru University, New Delhi, India).

### Iodixanol Density Gradient Centrifugation

The protein sample was overlaid on top of 10–40% discontinuous iodixanol (Sigma-aldrich, St. Louis, MO, United States) gradient and centrifuged in SW 55Ti rotor in an ultracentrifuge (Beckman Coulter, Indianapolis, IN, United States) for 3 h at 100,000 × *g* without braking. Ten equal fractions were collected from top and processed further, as indicated.

### Glycosidase Treatment

The purified protein was mixed with 10× glycoprotein denaturation buffer and incubated at 95°C for 5 min, chilled on ice and centrifuged for 10 s. Reaction mixture 2 (2 μl of 10× glycobuffer + 2 μl 10% NP40 + 6 μl H_2_O) was prepared and incubated at 37°C for 1 h. The denatured protein sample and reaction mixture 2 were mixed and 1 μl endoglycosidase H or PNGase F enzymes added and incubated for 4 h at 37°C. Aliquots of the samples were separated on SDS-PAGE and analyzed by Coomassie Brilliant Blue staining and western blot using anti-ORF2 antibody.

### Transmission Electron Microscopy

A Total of 5 μl of VLPs in suspension, at a concentration of 0.5 mg/ml, were adsorbed onto glow discharged Carbon-Formvar-coated copper grids for 2 min. The grids were then washed with PBS three times, followed by staining with 2% uranyl acetate. The grids were air-dried and examined in a Tecnai F20 electron microscope (FEI, Oregon, United States) operating at 200 kV.

### Hepatitis E Virus Patient Serum Analysis

To detect the ORF2 specific antibody in the HEV infected patients, western blotting was performed using sera from HEV patients and healthy individuals. Informed consent was obtained from the donors as per the institutional ethics committee guidelines. 1:20,000 dilution of serum was used and 1:10,000 dilution of goat anti-human IgG-HRP conjugated secondary antibody was used.

### Mice Experiments

The mice experiment protocol was duly approved by the Animal Ethics Committee of Translational Health Science and Technology Institute (THSTI), constituted under the provisions of CPCSEA (Committee for the Purpose of Control and Supervision on Experiments on Animals), Government of India. Animals were housed in the small animal facility of the THSTI and fed on standard pellet diet and water under pathogen-free conditions.

A total of eight groups of 6–8 week old male mice (*n* = 5) were immunized with 1 μg, 3 μg, and 5 μg ORF2 VLP in PBS or 1 μg, 3 μg, and 5 μg VLP emulsified with ALUM (1:1 volumetric ratio) by intraperitoneal route. Mice were boosted twice with the same dose of immunogens at 2 weeks interval. Two control groups were re-injected with PBS and PBS + ALUM, respectively. Blood samples were collected before each immunization and sera was prepared and stored at –80°C.

### Evaluation of Antigenicity

The titer of ORF2 specific IgG level in the serum obtained from each mouse at indicated time points was measured by ELISA. 96 well microtiter plates were coated with 100 ng purified 112–608 ORF2 protein in bicarbonate buffer for 16 h, followed by incubation with blocking buffer at 37°C for 2 h, as described in the method for ELISA. Subsequently, twofold serially diluted serum samples (in assay buffer) started at 1:100 were used as a primary antibody to analyze the antibody titer. Next, plates were incubated with HRP conjugated anti-rabbit IgG in assay buffer for 1 h at 37°C and washed three times in wash buffer. HRP activity was measured by colorimetry. Absorbance was measured at 450 nm.

### Cell Proliferation Assay

Cell proliferation assay was done with Cell Titer 96 Aqueous Non-Radioactive Cell Proliferation Assay Kit (Promega, Wisconsin, United States). Splenocytes were isolated from the spleen of the immunized and control mice on day 43 and cultured, as described previously (Kushwaha et al., 2012). Briefly, spleens were aseptically removed, gently macerated and passed through a sterile nylon cell strainer of 70 μm (BD Biosciences, California, United States). The cell suspension was centrifuged at 453 *g* in a swing-bucket rotor and the supernatant was discarded. Cells were resuspended in 0.84% chilled ammonium chloride solution (to lyse the erythrocytes), centrifuged at 453 *g* in a swing-bucket rotor and the supernatant was discarded. Next, cells were washed twice in RPMI medium, followed by resuspension in the RPMI medium containing 10% Foetal bovine serum (FBS). Cells were counted and seeded into 96-well plates at a density of 10 × 10^4^ cells/well and incubated at 37^0^C with 5% CO_2_. 24 h post-incubation, 5 μg of purified 112–608 ORF2 protein was added to the cultured cells. After 24 h, 20 μl MTS dye was added to each well and incubated for 4 h, followed by measurement of the absorbance at 490 nm. The proliferation was assessed by the stimulation index (SI), calculated according to the formula: SI = (experimental OD – control OD)/control OD.

### Statistics Analysis

Data are presented as mean ± standard errors of triplicate samples (SEM). Data are representative of two or more independent experiments. Data was analyzed using GraphPad Prism. Pairwise comparisons of values were performed using student’s *t*-test and multiple comparisons were analyzed by one-way ANOVA.

## Results

### Expression of 112–608aa ORF2 Protein in *Pichia pastoris*

**FIGURE 1:**
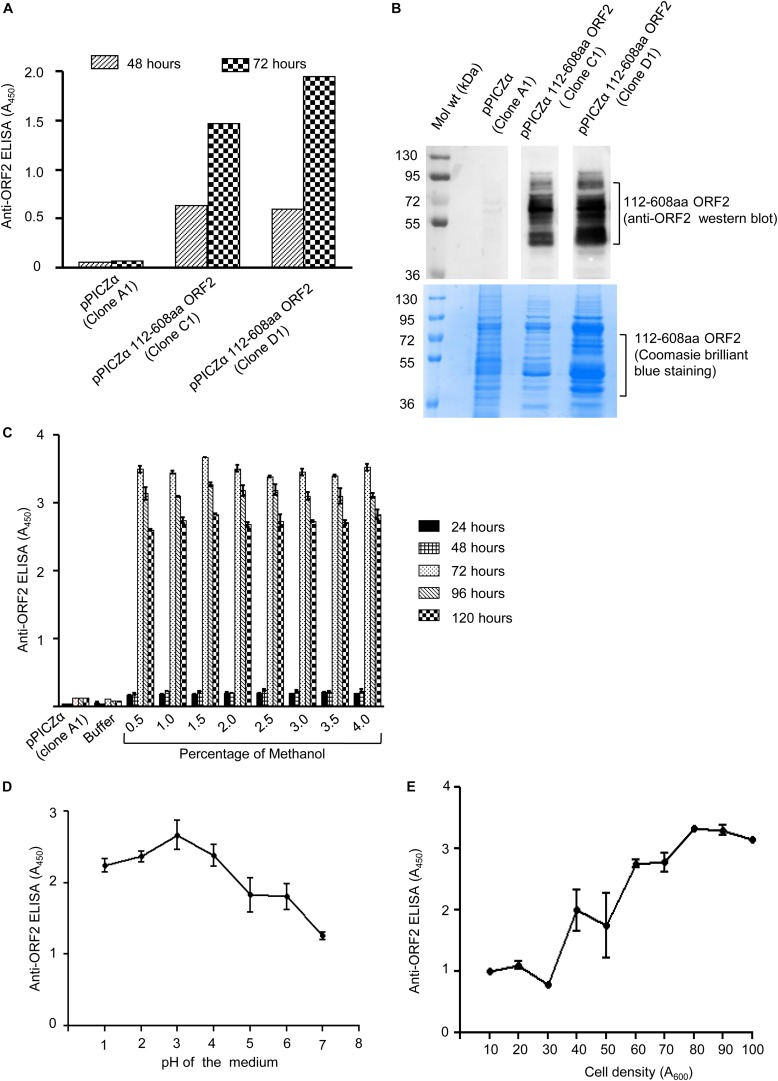
Expression of 112–608aa ORF2 in the *Pichia pastoris*. **(A)** ELISA of the *Pichia pastoris* secreted 112–608aa ORF2 protein in the supernatant of induced culture using anti-ORF2 antibody at indicated time points. **(B)** Upper panel: Western blot of the 48 h induced protein samples shown in **(A)** probed with anti-ORF2 antibody. Lower panel: Coomassie Brilliant Blue stained image of protein sample shown in upper panel **(C–E)**. ELISA of the culture medium of pPICZα 112–608aa ORF2 (clone D1) and pPICZα vector using anti-ORF2 antibody in following conditions. **(C)** Increasing methanol quantity and induction period (pH-3.0, OD_70_). **(D)** Increasing pH of the medium (72 h induction with 1% methanol at OD_70_). **(E)** Increasing cell density (72 h induction with 1% methanol at pH-3.0).

The two clones (clone C1 and D1) of pPICZα 112–608aa ORF2 and pPICZα vector were linearized using *Bst*XI enzyme and electroporated into *P. pastoris*, strain KM71H. Zeocin positive colonies were selected on YPDS medium supplemented with zeocin. To verify 112–608aa ORF2 expression, zeocin positive clones were grown in BMGY medium supplemented with 1% methanol. Level of 112–608aa ORF2 protein in the culture medium at different time point was measured by ELISA, which showed that clone D1 expressed more 112–608aa ORF2 compared to clone C1 (Figure 1A). Western blot of the culture medium using anti-ORF2 antibody confirmed the expression of 112–608aa ORF2 in both clones (Figure 1B upper panel). Coomassie brilliant blue staining of aliquots of the sample is shown in the lower panel (Figure 1B). Clone D1 was selected for optimization of culture parameters. ELISA data showed that the highest yield of 112–608aa ORF2 was obtained by 72 h incubation with 1.5% methanol (Figure 1C). pH analysis of the culture medium demonstrated that pH 3.0 is optimal for the maximum yield of the 112–608aa ORF2 (Figure 1D). Further, cell density of 80 (A_600_ = 80) favors maximum yield (Figure 1E).

### Purification of 112–608aa ORF2 Protein

**FIGURE 2:**
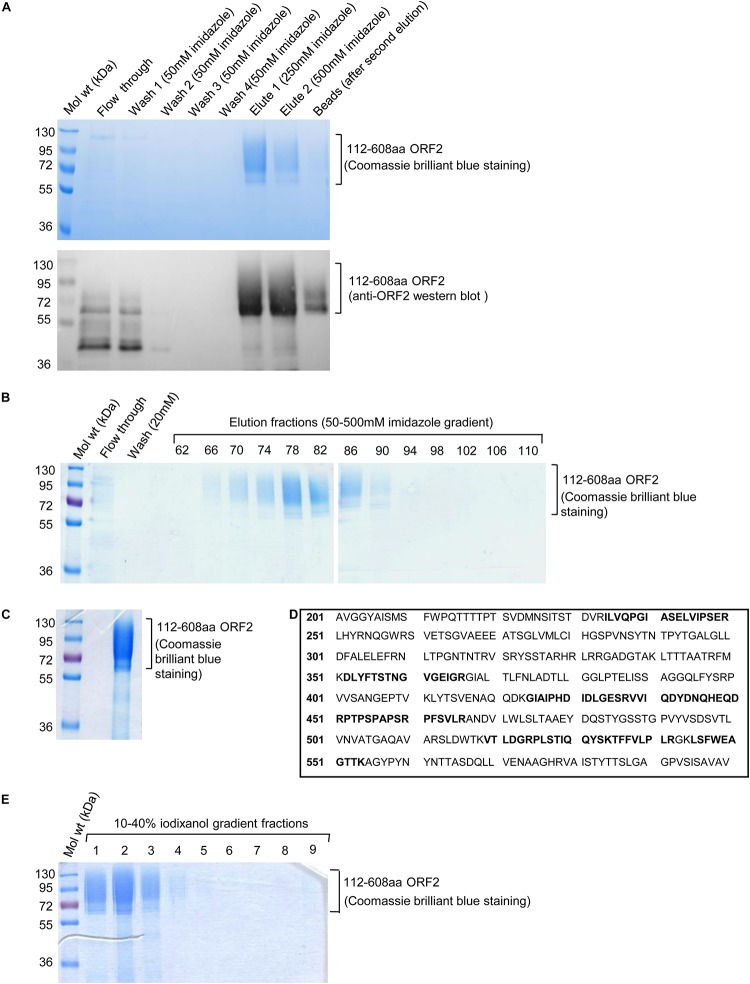
Ni-agarose and Ni-sepharose affinity purification and identification of 112–608aa ORF2 protein. **(A)** Upper panel: Coomassie Brilliant Blue stained image of the indicated fractions collected during batch purification of the recombinant 112–608aa ORF2 protein from the culture medium using Ni-agarose beads. Washing and elutions were performed with the indicated concentration of imidazole in 50 mM Tris buffer. 32 μl beads were boiled in laemelli buffer and loaded (beads); Lower panel: anti-ORF2 western blot of the samples shown in upper panel. **(B)** Coomassie Brilliant Blue stained image of the indicated fractions collected during FPLC purification of the 112–608aa ORF2 protein from the culture medium using Ni-Sepharose column. Elution was performed using 50–500 mM imidazole gradient. **(C)** Coomassie Briliant Blue stained image of the 112–608aa ORF2 protein obtained after buffer exchange of fractions 66–90 (shown in B) in PBS. **(D)** Identification of the 112–608aa ORF2 protein by mass spectrometry analysis. Peptides identified by mass spectrometry denoted as bold letters. **(E)** Coomassie Briliant Blue stained image of iodixanol gradient fractions, as indicated.

The culture media containing His-tagged 112–608aa ORF2 protein was incubated with Ni-agarose beads followed by removal of unbound protein by washing with 50 mm imidazole. Ni-agarose bound 112–608aa ORF2 was eluted in 250 mM and 500 mM imidazole (Figure 2A). Next, His-trap FF Ni-sepharose column was used to purify 112–608aa ORF2 from the culture medium by FPLC. Ni-sepharose bound 112–608aa ORF2 was eluted in a 50–500 mM imidazole gradient. The elution fractions showing strongest peak of protein were analyzed by SDS-PAGE and Coomassie brilliant blue staining, which showed that fraction 66–90 were enriched with the ORF2 protein (Figure 2B). These fractions were pooled and imidazole buffer was exchanged with PBS (pH 7.4) (Figure 2C). An aliquot of the protein was analyzed by MALDI-MS, which confirmed it to correspond to the HEV ORF2 protein (Figure 2D).

The purified protein was overlaid on top of 10–40% iodixanol gradient and subjected to ultracentrifugation. Total 10 fractions were collected from the top and analyzed by SDS PAGE followed by Coomassie Brilliant Blue staining. Fractions 1–3 were enriched with 112–608aa ORF2 protein (Figure 2E). These fractions were pooled and buffer exchanged with PBS (pH 7.4).

### Characterization of the Purified 112–608aa ORF2 Protein

**FIGURE 3:**
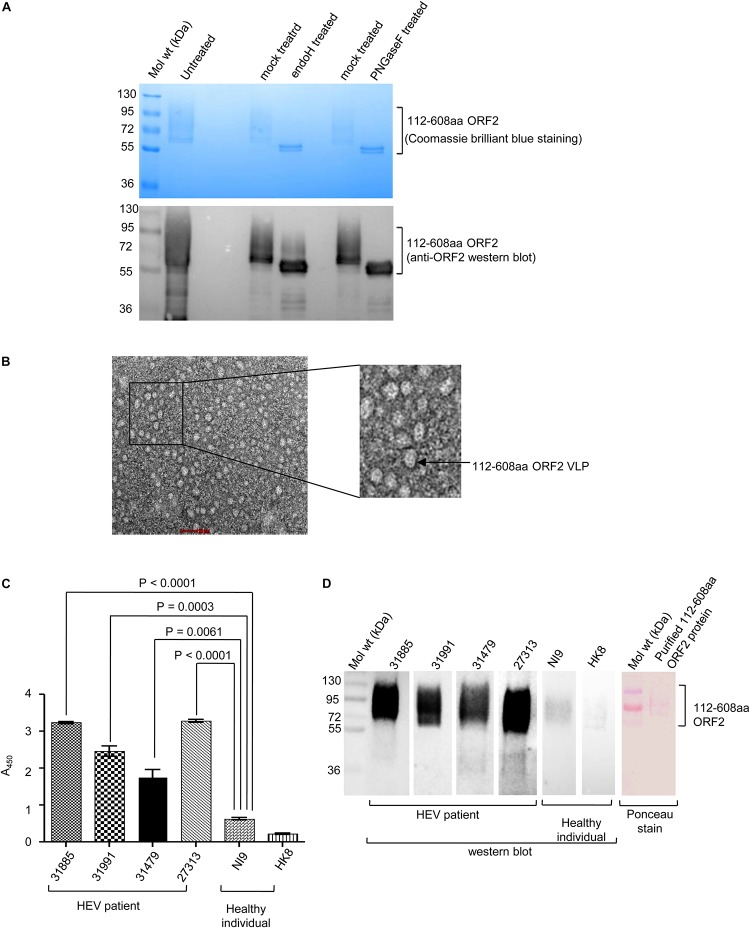
Characterization of the purified 112–608aa ORF2 protein. **(A)** Upper panel: Coomassie Brilliant Blue stained image of the glycosidase treated purified 112–608aa ORF2 protein. Lower panel: Western Blot of aliquots of samples shown in upper panel with anti-ORF2 antibody. **(B)** Transmission electron micrograph of the purified 112–608aa ORF2 protein (scale: 50 nm, magnification: 55000X). **(C)** ELISA of healthy controls and HEV patients sera using purified 112–608aa ORF2 protein as antigen. Data are mean ± SEM of triplicate samples. **(D)** Left panel: Western blot of the purified 112–608aa ORF2 using sera from the indicated samples; Right panel: Ponceau staining of a representative western blot.

Open reading frames 2 protein contains three N-linked glycosylation sites (Zafrullah et al., 1999). Susceptibility to deglycosylation enzymes, endoglycosidase H (endo H) and PNGase F was used to determine the glycosylation status of the purified protein Endo H cleaves the N-linked glycans between the two *N*-acetylglucosamine (GlcNAc) residues in the core region of the glycan chain on high-mannose glycans, leaving one GlcNAc still bound to the protein while PNGase F is a glycoamidase that cleaves the bond between the innermost GlcNAc and asparagine residues, releasing the entire sugar chain. Both Endo H and PNGase F could deglycosylate 112–608aa ORF2, as evident from Coomassie brilliant blue staining and anti-ORF2 western blot of the samples (Figure 3A). Further, whether the purified 112–608aa ORF2 protein could form VLP, was assessed by transmission electron microscopy (TEM). Although the population obtained was heterogeneous, a large proportion of particles of 22 nm diameter were clearly visible, suggesting that the purified 112–608aa ORF2 protein assembled into VLPs (Figure 3B).

Next, we evaluated if immunogenic epitopes were conserved in the purified 112–608aa ORF2 VLP. An ELISA was performed to measure the reactivity of the purified 112–608aa ORF2 VLP with anti-ORF2 antibody present in clinically confirmed HEV patient sera. As expected, HEV patient sera strongly interacted with the 112–608aa ORF2 VLP (Figure 3C). ELISA result was further confirmed by western blot of the 112–608aa ORF2 protein using the same sera. Significant reactivity was seen only in the presence of HEV patient sera and not in the sera from healthy individuals (Figure 3D).

### Induction of Humoral and Cellular Immune Response by 112–608aa ORF2 Protein

**FIGURE 4:**
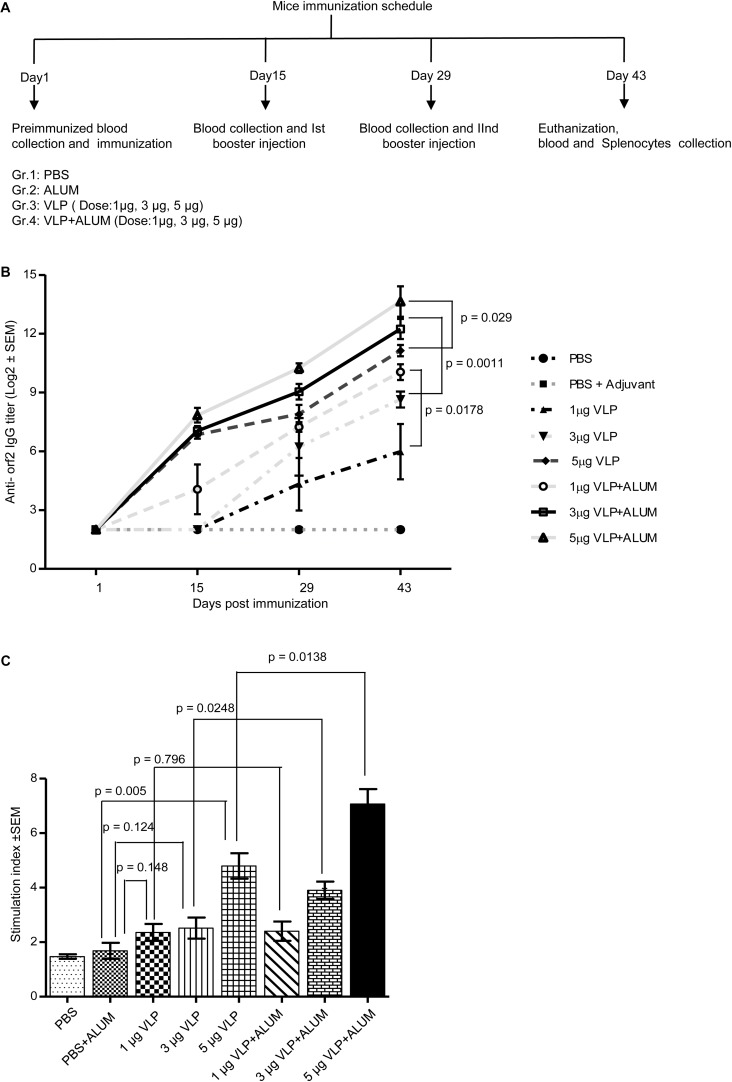
Evaluation of immunogenicity of the purified 112– 608aa ORF2 protein. **(A)** Schematic of mice immunization schedule. **(B)** Antibody titer profile of ORF2 specific IgG in the sera of mice immunized with purified 112–608aa ORF2 protein with or without ALUM, as indicated. The sera of all animals (*n* = 5) were collected before each immunization and twofold serially diluted starting from 1:100 for ELISA analysis. The reciprocal of the highest dilution showing two times absorbance of control mice was taken as positive antibody titer. Data represented as log2 transformed antibody titers and negative titer value was set as log value 2.0 for statistical analysis. **(C)** Cell proliferation assay of the cultured splenocytes harvested from the immunized mice, stimulated for 24 h with 5 μg purified 112–608aa ORF2 protein. The Stimulation index denotes the ratio of values obtained for immunized mice to that of the control mice. Data represented as mean ± SEM of 5 samples.

To evaluate the immunogenic potential of the 112–608aa ORF2 VLPs, immunization assay was performed in Balb/c mice. Six-week-old male mice were injected with the 112–608aa ORF2 VLPs, as illustrated (Figure 4A). Anti-ORF2 IgG titers were determined in serum by ELISA. Sera obtained from each mouse at indicated time points were twofold serially diluted starting from 1:100 and the reciprocal of the highest dilution that had two times absorbance of control mice was taken as positive ORF2-specific antibody titer. All the analysis was carried out on the log2 transformed antibody titers with standard error. The anti-ORF2 antibody titration shows that 112–608aa ORF2 VLPs induced ORF2 IgG production (Figure 4B). Alum emulsified 112–608aa ORF2 VLPs further increased the anti-ORF2 IgG level (Figure 4B). The IgG response was enhanced in a dose dependent manner, the titer being 1:12765 and 1:4837 for 5 μg VLP + ALUM and 3 μg VLP + ALUM samples, respectively.

The cellular immune response elicited by the 112–608aa ORF2 VLPs was evaluated by splenocytes proliferation assay. The splenocytes collected from 5 μgVLPs, 3 μgVLP + ALUM and 5 μgVLP + ALUM immunized mice could be significantly induced to proliferate, compared to controls (PBS/PBS + ALUM) (Figure 4C). The stimulation index of 3 μgVLP + ALUM (*p* = 0.0248) and 5 μgVLP + ALUM (*p* = 0.0138) groups were significantly higher, compared to only VLP, respectively (Figure 4C).

Taken together, our data shows that pichia expressed 112–608aa HEV ORF2 was glycosylated, formed VLPs and elicited significant immune response.

## Discussion

All four mammalian HEV genotypes show homology in the amino acid sequence of the capsid protein (ORF2), which has the capability to self assemble into VLPs. 60 copies of the 112–608aa ORF2 protein assemble to form the VLP (Zhang J.Z. et al., 2001; Xing et al., 2010). The three domains of ORF2, S (118-314), M (315-453), and P (454-606) play measure role in VLP formation. The S and M domains are highly conserved between genotypes and are the fundamental structural units in mature viral particles (Simanavicius et al., 2018). Epitope mapping studies demonstrate that monoclonal antibody binding sites are present on the S and M domain rather than the P domain (Meng et al., 2001; Zhang J.Z. et al., 2001).

The current cell culture system of HEV is not efficient enough to produce plenty of viruses for vaccination purpose (Tanaka et al., 2007). Moreover, recombinant ORF2 VLP also holds importance for developing diagnostic assays for HEV infection. Therefore, there is a lot of focus to produce HEV VLPs through recombinant means. Here, we used *P. pastoris* expression system to produce glycosylated 112–608aa ORF2 protein, which is secreted to the culture medium in the form of VLP. This VLP includes the S domain (absent in the 368–606aa ORF2 VLP currently used as HEV vaccine in China), which has been shown to be crucial for stabilization of the capsid shell (Xing et al., 2010).

Analysis of a limited number of HEV patient sera indicates that the 112–608aa VLPs retain the antigenic epitopes of the ORF2 protein. Since it is easy and economical to purify these VLPs, their diagnostic potential may be explored. Evaluation of immunogenicity of 112–608aa ORF2 VLPs in mice revealed that alum emulsified VLPs elicit stronger immune response compared to non-alum VLPs. Other adjuvants may be explored to identify the ideal combination formula.

Recently, it was reported that glycosylated and cleaved ORF2 proteins are most abundant in the infected patient sera and the same protein forms are highly recognized by patient antibodies (Montpellier et al., 2018; Ankavay et al., 2019). In this context, the 112–608aa ORF2 VLPs will be useful to evaluate the role of glycosylation status of ORF2 in mediating the immune response and protection from infection. Efforts are underway to generate non-glycosylated 112–608 ORF2 VLP and compare its protective efficacy to that of the glycosylated VLP. VLPs are considered to be good vaccine candidates, as they closely resemble native virus particles, without being infectious. Indeed, VLPs are being used as a licensed vaccine product for human papillomavirus (Olsson et al., 2007) and HBV (Murata et al., 2003). Therefore, the next step should focus on evaluating the efficacy of the *Pichia* expressed ORF2 VLPs in an infectious animal model of HEV. These VLPs may also be engineered to display additional antigenic epitopes from HEV or other pathogens. Future studies should aim at exploring such possibilities.

## Data Availability Statement

All datasets generated for this study are included in the article/supplementary material.

## Ethics Statement

The animal study was reviewed and approved by the Animal Ethics Committee of Translational Health Science and Technology Institute (THSTI), constituted under the provisions of CPCSEA (Committee for the Purpose of Control and Supervision on Experiments on Animals), Government of India.

## Author Contributions

JG and MS contributed to the experimental design and data analysis. JG, SK, and MS wrote the manuscript. JG, SK, AS, SG, and CS performed the experiments. MB, Shalimar, BN, GB, NK, and CR-K provided material, analysis tools, and suggestions.

## Conflict of Interest

The authors declare that the research was conducted in the absence of any commercial or financial relationships that could be construed as a potential conflict of interest.
